# Breast Cancer and Risk of Depression: A Comparative Cross-Sectional Study Among Women With and Without Breast Cancer in Addis Ababa, Ethiopia

**DOI:** 10.1200/GO.24.00235

**Published:** 2024-10-10

**Authors:** Alem Gebremariam, Adamu Addissie, Nebiyu Dereje, Mathewos Assefa, Ahmedin Jemal

**Affiliations:** ^1^ Department of Public Health, College of Medicine and Health Sciences, Adigrat University, Adigrat, Ethiopia; ^2^ Tigray Health Research Institute, Mekelle, Ethiopia; ^3^ Global Health Working Group, Martin-Luther-University, Halle-Wittenberg, Halle, Germany; ^4^ School of Public Health, College of Health Sciences, Addis Ababa University, Addis Ababa, Ethiopia; ^5^ Department of Public Health, College of Medicine and Health Sciences, Wachemo University, Hossana, Ethiopia; ^6^ Radiotherapy Center, School of Medicine, Addis Ababa University, Addis Ababa, Ethiopia; ^7^ Surveillance and Health Equity Science, American Cancer Society, Atlanta, GA

## Abstract

**PURPOSE:**

The extent of symptoms of depression among patients with breast cancer compared with those without the disease is not well documented in Ethiopia and other sub-Saharan African countries.

**MATERIALS AND METHODS:**

This study examines the prevalence of symptoms of depression in women with breast cancer (n = 436) compared with those without breast cancer (n = 856) in Addis Ababa, Ethiopia, through a comparative cross-sectional study using a validated questionnaire, the Patient Health Questionnaire-9. The association between breast cancer diagnosis and symptoms of depression was evaluated using a multivariable binary logistic regression model.

**RESULTS:**

About 39.2% of women with breast cancer had some level of symptoms of depression compared with 23.8% of women without the disease. By severity of symptoms of depression, 13.1% of women with breast cancer reported moderate to severe symptoms of depression compared with 6.8% of women without the disease. Sixty-three percent of women with breast cancer reported difficulties performing routine daily activities, compared with 36.7% of women without the disease. In the multivariable-adjusted model, women with breast cancer were 2 times (adjusted odds ratio, 2.26 [95% CI, 1.49 to 3.44]) more likely to report symptoms of depression compared with those without the disease. Likewise, women with breast cancer were 4.78 (95% CI, 3.51 to 6.52) times more likely to report difficulty in performing routine daily activities compared with women without the disease.

**CONCLUSION:**

Four in 10 women with breast cancer in Addis Ababa reported having symptoms of depression, which was considerably higher than women in the general population. This finding emphasizes the importance of addressing psychosocial needs among women with breast cancer to enhance quality of life and potentially extend longevity.

## Teaser

Breast cancer doubles depression risk and severely impacts the daily lives of women in Ethiopia.

## INTRODUCTION

Depression is a common mental disorder in Ethiopia.^1,2^ According to a 2018 study by Bifftu et al,^1^ 20.5% of individuals in the general population reported depression in the country, with only 38% of individuals seeking medical help. The prevalence of depression among women with breast cancer is likely to be greater than those reported in the general population. For instance, Belay et al documented that more than half (58.6%) of the women with breast cancer at Tikur Anbessa Specialized Hospital in Addis Ababa reported symptoms of depression.^3^ Nevertheless, there are limited data on the level of symptoms of depression in women with breast cancer compared with those without the disease in Ethiopia or other sub-Saharan countries. Herein, we conducted a comparative cross-sectional study to estimate the burden of symptoms of depression associated with breast cancer among women in Addis Ababa, Ethiopia.

**CONTEXT**

**Key Objective**
To assess the risk of symptoms of depression among women with breast cancer compared with women without breast cancer in Addis Ababa, Ethiopia.
**Knowledge Generated**
In a comparative analysis of symptoms of depression burden in women with and without breast cancer, our research revealed that those with breast cancer were 2.5 times more likely to experience symptoms of depression and five times more likely to feel difficulties performing their daily activities compared with those without the illness.
**Relevance**
The study highlights the urgent need for enhanced psychosocial support services for women with breast cancer to mitigate the effect of depression on their well-being.

## MATERIALS AND METHODS

### Design, Participants, and Procedure

A comparative cross-sectional study was conducted among 441 women newly diagnosed with breast cancer from January 1, 2017, to June 30, 2018, residing in Addis Ababa city and receiving care from seven public and private health facilities, and 882 women randomly selected from the city. Five women with breast cancer were excluded from the study due to missing data for symptoms of depression. Likewise, 26 women among the comparison group were excluded because they refused to participate in the study (21 women) or had a history of cancer (five women). The final analysis was based on 436 women with breast cancer and 856 women without a history of cancer. More details on the study design and participants' sociodemographic characteristics are given in Gebremariam et al.^4^

### Variables Measurement

The primary outcome of the study, symptoms of depression, was measured using a validated tool,^5-7^ the Patient Health Questionnaire-9 (PHQ-9), through face-to-face interviews. The reliability of the items was assessed using Cronbach's alpha, which was .81, with item levels ranging from .78 to .82. Participants were asked whether they had experienced symptoms of depression within the last 2 weeks from the date of the interview on the basis of nine Likert-scale questions with responses ranging from zero (not at all) to three (nearly every day). The scores (responses) from the nine Likert-scale questions were summed up to determine the degree of symptoms of depression, ranging from 0 to 27, with larger scores indicating greater degree of symptoms of depression. Following Wondimagegnehu et al’s^8^ approach, the scores were grouped into five levels of symptoms of depression: none or minimal (0-4), mild (5-9), moderate (10-14), moderately severe (15-19), and severe (20-27). In addition, we dichotomized symptoms of depression scores into mild or low level of symptoms of depression (<10) and moderate or high level of symptoms of depression (≥10) to further facilitate interpretation of findings.

Level of difficulty in performing routine daily activities, the secondary outcome of interest, was measured as not difficult at all, somewhat difficult, very difficult, and extremely difficult. For further analysis in the logistic regression, this was dichotomized into binary variable as No if the response was not difficult at all or Yes otherwise.

Breast cancer status, the primary exposure variable, was categorized as Yes for women newly diagnosed with breast cancer recruited from cancer treatment centers and as No for women without a history of cancer and recruited from the general population. Similarly, self-reported sociodemographic variables, including age, marital status, education, occupation, and income, were categorized ordinally or nominally for inclusion in the multivariable logistic regression model to determine the association between symptoms of depression and breast cancer status.

### Statistical Methods

Data were entered into Epi-Info and exported to Stata version 14 (STATA Corporation, Houston, TX) for analysis. The data were analyzed descriptively and then through binary logistic regression to evaluate the adjusted association between breast cancer status and having a moderate or higher level of symptoms of depression as well as the level of difficulty in performing routine daily activities. Variables with a *P* < .25 in the bivariate association and those with clinical relevance were included in the multivariable binary logistic regression. The adjusted odds ratios (AORs) with their 95% CIs were calculated, and statistical significance was declared at a *P* < .05. Ethical clearance was obtained from the Institutional Review Board of the School of Public Health of Addis Ababa University. Oral consent to participate in the study was obtained from the respondents. Confidentiality and anonymity were maintained throughout the study.

### Ethical Approval

This study received ethical approval from the Institutional Review Board of the College of Health Sciences, Addis Ababa University (018/17/SPH). Participants provided verbal consent to take part in the study after being informed about it.

### Informed Consent

Informed consent was obtained from all individual participants included in the study.

## RESULTS

The median age (IQR) of women with and without cancer was 40 (35-55) years and 37 (30-48) years, respectively. Women with breast cancer were more likely to have higher educational attainment and be older. For example, 55% of women with breast cancer had a secondary or higher level of education, compared with 42.6% of those without breast cancer (Table 1).

**TABLE 1:** Sociodemographic and Clinical Characteristics of Women With and Without Breast Cancer in Addis Ababa, Ethiopia, 2019 (N = 1,292)

Women Characteristics	Women With BC (n = 436), No. (%)	Women Without BC (n = 856), No. (%)	*P*
Age group in years			
≤30	54 (12.4)	251 (29.3)	<.001
31-39	129 (29.6)	244 (28.5)	
40-49	105 (24.1)	152 (17.8)	
≥50	148 (33.9)	209 (24.4)	
Currently married			
Yes	252 (57.8)	520 (60.7)	.307
No	184 (42.2)	336 (39.3)	
Educational attainment			
Not attended school	85 (19.5)	237 (27.7)	<.001
Primary school	111 (25.5)	255 (29.8)	
Secondary school	140 (32.1)	217 (25.3)	
Diploma or above	100 (22.9)	147 (17.2)	
Occupation			
Homemaker	213 (48.9)	422 (49.3)	.775
Government employee	61 (14.0)	109 (12.7)	
Private employee^a^	136 (31.2)	282 (33.0)	
Others^b^	26 (5.9)	43 (5.0)	
Family size			
1-2	64 (14.7)	83 (9.9)	.027
3-5	256 (58.7)	539 (64.4)	
≥6	116 (26.6)	215 (25.7)	
Family monthly income, $			
<$61 USD	121 (27.8)	392 (45.8)	<.001
$61.0-194.0 USD	199 (45.6)	269 (31.4)	
$>194.0 USD	106 (24.3)	115 (13.4)	
Do not know	10 (2.3)	80 (9.4)	

Abbreviation: NGO, nongovernmental organization.

^a^ Merchant, daily laborer, NGO worker.

^b^ Jobless, students, retirees.

Table 2 shows the levels of symptoms of depression and difficulties in performing routine daily activities. Symptoms of depression were notably higher in women with breast cancer compared with those without it. Among women with breast cancer, the median (IQR) of the sum score of symptoms of depression was three (1-7), whereas for women without breast cancer, it was 0 (0-4). Additionally, the prevalence of moderate or higher symptoms of depression was significantly greater among women with breast cancer (13.1%; 95% CI, 10.2 to 16.5) compared with those without it (6.8%; 95% CI, 5.3 to 8.6). When asked about their ability to perform routine daily activities such as managing household responsibilities and maintaining relationships, 72.9% of women with breast cancer reported some level of difficulties compared with only 26% of women without the disease.

**TABLE 2:** Overall PHQ-9 Score and Level of Difficulties Experienced by Women With and Without Breast Cancer in Addis Ababa, Ethiopia, 2019 (N = 1,292)

Variable	Women With Breast Cancer (n = 436), No. (%)	Women Without Breast Cancer (n = 856), No. (%)	*P*
Overall median (IQR) of the score	3 (1-7)	0 (0-4)	
Severity of symptoms of depression			<.001
None or minimal (0-4)	265 (60.8)	652 (76.2)	
Mild (5-9)	114 (26.1)	146 (17.0)	
Moderate (10-14)	47 (10.8)	46 (5.4)	
Moderately severe (15-19)	9 (2.1)	8 (1.0)	
Sever (20-27)	1 (0.2)	4 (0.4)	
Symptoms of depression (moderate and above)			<.001
No (PHQ-9 ≤9 score)	379 (86.9)	798 (93.2)	
Yes (PHQ-9 ≥10 score)	57 (13.1)	58 (6.8)	
Level of difficulties in performing routine daily activities			<.001
Not difficult at all	96 (27.1)	381 (63.3)	
Somewhat difficult	216 (60.8)	148 (24.6)	
Very difficult	40 (11.3)	53 (8.8)	
Extremely difficult	3 (0.8)	20 (3.3)	

Abbreviation: PHQ-9, Patient Health Questionnaire-9.

Table 3 depicts the multivariable-adjusted and unadjusted associations between symptoms of depression and breast cancer status. Women with breast cancer had approximately two times higher odds of symptoms of depression (AOR, 2.26 [95% CI, 1.49 to 3.44]) compared with those without the disease. Additionally, women with no formal schooling had greater odds of symptoms of depression (AOR, 3.40 [95% CI, 1.33 to 8.68]) than those with a higher education level (diploma and above; Table 3).

**TABLE 3:** Adjusted Association Between Women's BC Status and Symptoms of Depression in Addis Ababa, Ethiopia, 2019 (N = 1,292)

Demographic Characteristics	Symptoms of Depression		Unadjusted Odds Ratio (95% CI)	Adjusted Odds Ratio (95% CI)
	Yes, No. (%)	No, No. (%)		
Cancer status				
Women without BC	58 (6.8)	798 (93.2)	Ref.	Ref.
Women with BC	57 (13.1)	379 (86.9)	2.06 (1.40 to 3.04)***	2.26 (1.49 to 3.44)***
Age group in years				
≤30	19 (6.2)	286 (93.8)	Ref.	Ref.
31-39	28 (7.5)	345 (92.5)	1.22 (0.66 to 2.23)	1.11 (0.59 to 2.07)
40-49	26 (10.1)	231 (89.9)	1.69 (0.91 to 3.13)	1.39 (0.73 to 2.64)
≥50	42 (11.8)	315 (88.2)	2.00 (1.14 to 3.53)*	1.35 (0.72 to 2.52)
Marital status				
No	57 (11.0)	463 (89.0)	Ref.	Ref.
Yes	58 (7.5)	714 (92.5)	0.65 (0.44 to 0.96)*	0.80 (0.53 to 1.23)
Education				
Diploma or above	7 (2.8)	240 (97.2)	Ref.	Ref.
Secondary school	32 (9.0)	325 (91.0)	3.37 (1.46 to 7.77)**	2.77 (1.16 to 6.64)*
Primary school	39 (10.7)	327 (89.3)	4.08 (1.79 to 9.29)**	3.29 (1.35 to 7.99)**
Not attended school	37 (11.5)	285 (88.5)	4.45 (1.94 to 10.16)***	3.40 (1.33 to 8.68)*
Occupation				
Homemaker	60 (9.5)	575 (90.6)	Ref.	Ref.
Government employee	8 (4.7)	162 (95.3)	0.47 (0.22 to 1.00)	0.79 (0.35 to 1.80)
Private employee^a^	39 (9.3)	379 (90.7)	0.98 (0.64 to 1.50)	1.15 (0.73 to 1.82)
Others^b^	8 (11.6)	61 (88.4)	1.25 (0.57 to 2.75)	1.35 (0.59 to 3.09)
Family monthly income, $				
$<61 USD	52 (10.1)	461 (89.9)	Ref.	Ref.
$61.0-194.0 USD	48 (10.3)	420 (89.7)	1.01 (0.66 to 1.53)	1.11 (0.70 to 1.76)
$>194.0 USD	8 (3.6)	213 (96.4)	0.33 (0.15 to 0.71)**	0.47 (0.20 to 1.09)
Do not know	7 (7.8)	83 (92.2)	0.74 (0.32 to 1.70)	1.00 (0.43 to 2.33)

Abbreviations: BC, breast cancer; NGO, nongovernmental organization; Ref, reference group.

^a^ Merchant, daily laborer, NGO worker.

^b^ Jobless, students, pensioned, commercial sex worker, volunteer, and aid-dependent.

*P < .05; **P < .01; ***P < .001.

Table 4 presents the adjusted and unadjusted associations between breast cancer status and difficulties in performing routine daily activities. Women diagnosed with breast cancer had five times higher odds of difficulties in performing daily activities (AOR, 4.78 [95% CI, 3.51 to 6.52]) compared with those without the condition. Also, women with primary school education had higher odds of difficulties in performing routine daily activities (AOR, 1.82 [95% CI, 1.12 to 2.96]) than those with a diploma or higher education.

**TABLE 4:** Adjusted Association Between Women's BC Status and Difficulty Performing Daily Activities in Addis Ababa, Ethiopia, 2019

Women Characteristics	Having Difficulty in Performing Daily Activities		Unadjusted Odds Ratio (95% CI)	Adjusted Odds Ratio (95% CI)
	Yes, No. (%)	No, No. (%)		
Cancer status				
Women without BC	221 (36.7)	381 (63.3)	Ref.	Ref.
Women with BC	257 (73.0)	96 (27.0)	4.65 (3.49 to 6.19)***	4.78 (3.51 to 6.52)***
Age group in years				
≤30	91 (42.9)	121 (57.1)	Ref.	Ref.
31-39	125 (46.1)	146 (53.9)	1.13 (0.79 to 1.63)	0.80 (0.53 to 1.19)
40-49	113 (58.6)	80 (41.4)	1.87 (1.26 to 2.78)**	1.29 (0.84 to 1.98)
≥50	151 (53.7)	130 (46.3)	1.54 (1.07 to 2.21)*	1.04 (0.68 to 1.59)
Currently on marriage				
No	206 (50.5)	202 (49.5)	Ref.	Ref.
Yes	274 (49.9)	275 (50.1)	0.97 (0.75 to 1.26)	1.03 (0.76 to 1.39)
Educational attainment				
Diploma or above	79 (45.4)	95 (54.6)	Ref.	Ref.
Secondary school	131 (51.2)	125 (48.8)	1.26 (0.85 to 1.85)	1.41 (0.88 to 2.23)
Primary school	145 (53.1)	128 (46.9)	1.36 (0.93 to 1.95)	1.82 (1.12 to 2.96)*
Not attended school	125 (49.2)	129 (50.8)	1.16 (0.79 to 1.71)	1.63 (0.95 to 2.79)
Occupation				
Homemaker	248 (50.7)	241 (49.3)	Ref.	Ref.
Government employee	57 (47.5)	63 (52.5)	0.87 (0.58 to 1.31)	0.97 (0.59 to 1.57)
Private employee^a^	149 (50.0)	149 (50.0)	0.97 (0.72 to 1.29)	1.12 (0.81 to 1.56)
Others^b^	26 (52.0)	24 (48.0)	1.05 (0.58 to 1.88)	1.14 (0.59 to 2.21)
Family monthly income, $				
$<61 USD	183 (46.3)	212 (53.7)	Ref.	Ref.
$61.0-194.0 USD	193 (54.5)	161 (45.5)	1.38 (1.04 to 1.85)*	1.21 (0.86 to 1.70)
>$194.0 USD	81 (52.6)	73 (47.4)	1.28 (0.88 to 1.86)	1.18 (0.72 to 1.93)
Do not know	23 (42.6)	31 (57.4)	0.85 (0.48 to 1.52)	1.10 (0.59 to 2.03)

Abbreviations: BC, breast cancer; NGO, nongovernmental organization; Ref, reference group.

^a^ Merchant, daily laborer, NGO worker.

^b^ Jobless, students, pensioned, commercial sex worker, volunteer, and aid-dependent.

*P < .05; **P < .01; ***P < .001.

## DISCUSSION

On the basis of a comparative cross-sectional study of women with and without breast cancer in Addis Ababa, we found that women with breast cancer were more than twice as likely to report overall and severe symptoms of depression compared with women without the disease. Furthermore, women with breast cancer were five times more likely to report difficulties in carrying out routine daily activities. We could not find similar published data in Ethiopia or other sub-Saharan African countries to discuss our findings in relation to the existing literature. However, our finding of 39.2% of women with breast cancer reporting symptoms of depression was considerably lower than that reported by Ayalew et al^9^ among women with breast cancer seen at the Tikur Anbessa Teaching Hospital (62.8%) but much higher than that reported by Wondimagegnehu et al^8^ in the same hospital (25%). Also, several studies outside of Africa reported a considerable proportion of women experience difficulties in carrying routine daily activities and loss of productivity.^10,11^ For example, a systematic review conducted by Neo et al^12^ found that more than half (54.6%) of the adults with cancer encountered difficulties in performing routine daily activities, and Kim et al^13^ reported that 66.1% of women diagnosed with breast cancer in Korea experienced moderate to severe fatigue.

Furthermore, several studies noted that women with breast cancer alongside depression face greater impairment and reduced access to social support,^8,14^ lower quality of life,^15^ reduced treatment adherence,^16^ recurrence of breast cancer following treatment,^17,18^ increased health care costs,^16^ and poor survival.^17,19^ For example, a meta-analysis conducted by Wang et al^17^ revealed that patients with breast cancer with depression had a roughly 30% higher risk of all-cause and cancer-specific mortality (hazard ratio [HR], 1.29 [95% CI, 1.11 to 1.49]) compared with those without depression. Similarly, a study conducted by Walker et al^20^ in the United Kingdom showed that women diagnosed with breast cancer and severe depression experienced a 42% higher risk of mortality (HR, 1.42 [95% CI, 1.15 to 1.75]) than their counterparts.

Our finding of higher risk of symptoms of depression in women with lower levels of education is consistent with previous studies conducted in Ethiopia^21^ and other countries.^22,23^ This might be due to individuals with lower educational status having less opportunities to be aware of their disease condition and finding different coping mechanisms to deal with their situation.^24-26^

Our study found that women with breast cancer are two times more likely to report symptoms of depression and five times more likely to face difficulties in daily activities. Despite the high prevalence of symptoms of depression and its negative effect, there is a low rate of health service attendance among those with depressive episodes, with lower educational status being a barrier to seeking treatment among the general population,^21^ and depression is often underdiagnosed and undertreated in patients with cancer as well.^27,28^ However, the use of brief screening questionnaires, including the PHQ-9, is valid in primary care settings in Ethiopia.^29^ This highlights the need to incorporate screening for depression^30^ and psychosocial therapies^31^ into standard cancer care to address the mental health needs of Ethiopian women with breast cancer, not just to enhance their quality of life but also to potentially extend their lifespan.

In general, the significantly higher rates of symptoms of depression in women with breast cancer in Addis Ababa could be attributed to the following contextual factors in the country. First, the health care infrastructure for cancer care and other specialized care is limited.^32^ For instance, at the time of the assessment, there was only one radiotherapy machine in the country and patients had to wait for over a year for receipt of radiation.^32^ Second, cancer accentuates the already prevailing poverty^33^—resulting in the inability to pay for their cancer treatment and care.^34^ Third, the community's cultural beliefs (eg, cancer as a death sentence) and stigma surrounding both cancer and mental health issues^35^ can prevent individuals from seeking medical help for depression.^36^ Finally, there are no well-structured psychological support systems in the cancer treatment centers in the country.^8,37^ Addressing these factors through improved health care infrastructure, education, and provision of culturally tailored psychosocial supports can help mitigate the burden of depression in women with breast cancer.

A strength of our study is to report the extent of symptoms of depression in women with breast cancer in relation to women without the disease in Addis Ababa–based, African setting. Our study, however, has the following limitations. First, there is a risk of social desirability bias, in which patients may either exaggerate or minimize the symptoms of depression and anxiety for some reason although this bias likely to be nondifferential. Second, the findings may not be generalizable to patients with breast cancer outside of Addis Ababa, especially in rural parts of Ethiopia. Third, symptoms of depression were self-reported and were not diagnostically confirmed.

In conclusion, four in 10 women with breast cancer in Addis Ababa reported having symptoms of depression, which was considerably higher than women in the general population. This finding emphasizes the importance of addressing psychosocial needs among women with breast cancer to enhance quality of life and potentially extend longevity.

## Data Availability

The data sets used and/or analyzed during the current study are available from the corresponding author upon reasonable request (alemg25@gmail.com).
